# Agonistic and antagonistic targeting of immune checkpoint molecules differentially regulate osteoclastogenesis

**DOI:** 10.3389/fimmu.2023.988365

**Published:** 2023-02-02

**Authors:** Victoria C. Brom, Andreas C. Strauss, Alexander Sieberath, Jochen Salber, Christof Burger, Dieter C. Wirtz, Frank A. Schildberg

**Affiliations:** ^1^ Clinic for Orthopedics and Trauma Surgery, University Hospital Bonn, Bonn, Germany; ^2^ Department of Experimental Surgery, Centre for Clinical Research, Ruhr-Universität Bochum, Bochum, Germany; ^3^ Department of Surgery, Universitätsklinikum Knappschaftskrankenhaus Bochum GmbH, Bochum, Germany

**Keywords:** immune checkpoint molecules, osteoclasts, osteolysis, osteoporosis, endoprosthesis loosening, cancer, osteoimmunology, musculoskeletal immunology

## Abstract

**Introduction:**

Immune checkpoint inhibitors are used in the treatment of various cancers and have been extensively researched with regard to inflammatory and autoimmune diseases. However, this revolutionary therapeutic strategy often provokes critical auto-inflammatory adverse events, such as inflammatory reactions affecting the cardiovascular, gastrointestinal, nervous, and skeletal systems. Because the function of these immunomodulatory co-receptors is highly cell-type specific and the role of macrophages as osteoclast precursors is widely published, we aimed to analyze the effect of immune checkpoint inhibitors on these bone-resorbing cells.

**Methods:**

We established an *in vitro* model of osteoclastogenesis using human peripheral blood mononuclear cells, to which various immune checkpoints and corresponding antagonistic antibodies were administered. Formation of osteoclasts was quantified and cell morphology was analyzed *via* immunofluorescence staining, cell size measurements, and calculation of cell numbers in a multitude of samples.

**Results:**

These methodical approaches for osteoclast research achieved objective, comparable, and reproducible results despite the great heterogeneity in the form, size, and number of osteoclasts. In addition to the standardization of experimental analyses involving osteoclasts, our study has revealed the substantial effects of agonistic and antagonistic checkpoint modulation on osteoclastogenesis, confirming the importance of immune checkpoints in bone homeostasis.

**Discussion:**

Our work will enable more robust and reproducible investigations into the use of immune checkpoint inhibitors in conditions with diminished bone density such as osteoporosis, aseptic loosening of endoprostheses, cancer, as well as the side effects of cancer therapy, and might even pave the way for novel individualized diagnostic and therapeutic strategies.

## Introduction

Conditions characterized by an imbalance in bone homeostasis cause a great deal of suffering for patients. Unfortunately, it is not fully known what causes the dysregulation of osteoclasts (OCs) and osteoblasts (OBs), which leads to impaired bone quality. The current diagnostic and therapeutical management for diseases such as osteoporosis, aseptic loosening of endoprostheses, and certain cancers is insufficient. However, there is evidence about underlying osteoimmunological mechanisms that are immensely relevant in development of osteoporotic conditions and skeletal fractures (1–7).

Immune checkpoint molecules represent promising immunological targets in a number of disease settings, and are widely expressed on multiple cell types. They function as secondary regulators for communication between antigen-presenting cells (APCs) and T cells, that is crucial for sufficient immune responses to pathogenic cells. Interestingly, immune checkpoints modulate this interaction not only as co-stimulatory but also as co-inhibitory receptors (8, 9). Administration of inhibitory antibodies targeting such immune checkpoint molecules leads to improved immune activation against various cancers and other diseases (10–12) rather than direct elimination of pathogens (13–16). Stimulatory and inhibitory checkpoint molecules can both be either strengthened or suppressed, resulting in a complex immunoregulatory concept.

Consequently, broad spectrum immune checkpoint inhibitors (ICI) are already successfully used in treatment of various cancers (17, 18), however, patients present side effects arising from excessive autoimmunity and the induction of inflammatory adverse events (19–22). Incidence and severity of such side effects vary considerably depending on the disease, the targeted checkpoint molecule, the applied concentration, single or combined application and patient constitution (20, 21, 23–26). Still, ICI represents a highly potent approach particularly for cancer diseases, that offers immense value for patients and could merely be optimized by amelioration of adverse event occurrence. For this reason, increasing studies suggest the use of cell-type specific checkpoint modulation, as such an approach not only provides more effective immunity against the specific disease but is also more likely to minimize immune-related side effects (27–29).

It has been shown that checkpoint molecules regulate myeloid cells (30–33). Myeloid cells such as monocytes and macrophages are promising targets for the treatment of cancer, as well as infectious and autoimmune diseases. Some of these diseases are caused by low levels of checkpoint protein expression, instead of checkpoint overexpression, meaning that stimulation with agonistic antibodies represents a promising therapeutical approach (19). Monocytes and macrophages also play a critical role in bone homeostasis as they function as precursor cells for OC maturation. We thus sought to examine the influence of various immune checkpoint proteins and their antagonistic antibodies on osteoclastogenesis to assess whether and which checkpoint molecules alter OC differentiation and may be responsible for OC overactivation that ultimately disrupts bone homeostasis. In this regard, some results for few checkpoint molecules are already published, however, mostly describe controverse findings.

Our primary study goal was therefore to gain a deeper understanding of bone homeostasis regulation and elucidate whether and how immune checkpoint pathways could be exploited to improve the diagnosis and treatment of conditions that lead to bone loss.

## Materials and methods

### OC differentiation

In preparation for the differentiation of osteoclasts, purification of human peripheral blood mononuclear cells (PBMC) was performed. Whole blood samples were collected in lithium heparin tubes (SARSTEDT AG & Co. KG, Nümbrecht, Germany), diluted with equal amounts of phosphate buffered saline (PBS; Thermo Fisher Scientific, Waltham, MA, USA), and gently added to the same volume of BioColl (Bio & Sell, Feucht, Germany) in a 50-mL Falcon tube. The tubes were then centrifuged (800 g, 4°C, 25 min, brake off). The interphase cell layer was transferred to a new 50-mL Falcon tube and resuspended in enough PBS (4°C) to fill the tube, prior to being centrifuged again (800 g, 4°C, 15 min, brake on). After discarding the supernatant, the cell pellet was washed twice with PBS and centrifuged (230 g, 4°C, 5 min, brake on). Subsequently, the resulting cell pellet was resuspended in Minimum Essential Medium α (MEMα; Thermo Fisher Scientific, Waltham, MA, USA) containing 20% heat-inactivated fetal bovine serum (FBS; Sigma-Aldrich, St. Louis, MO, USA), 2 g/L HEPES (Carl Roth, Karlsruhe, Germany), 1% L-glutamine (Thermo Fisher Scientific, Waltham, MA, USA), 1% penicillin-streptomycin (PS; Thermo Fisher Scientific, Waltham, MA, USA). The cell suspension was then plated into 12-well Ibidi slides at 500,000 cells per well (0.56 cm^2^, removable silicon chamber, Ibidi, Gräfelfing, Germany). Differentiation of mature OCs was initiated on day 2 by adding 50 ng/mL M-CSF, 50 ng/mL RANK-Ligand (RANKL; Miltenyi, Bergisch Gladbach, Germany), and 10 nM 1α,25-Dihydroxyvitamin D3 (Sigma-Aldrich, St. Louis, MO, USA) to the standard medium. Differentiation medium was changed every 48 – 72h. On day 2, the reagents indicated in **Supplementary Table 1** were added to the differentiation medium for investigation of their effects on osteoclastogenesis as well as with every further medium change. Additional control experiments were performed using application of non-binding unspecific antibodies and proteins to ensure validity of the non-treated OC control (**Supplementary Figure 1**). The ethics committee of the University of Bonn, Germany, approved the study (approval code 283/21), which was conducted according to the approved guidelines and the Helsinki Declaration.

### Validation of OC differentiation

On day 14 of OC culture, cells were fixed using paraformaldehyde 4% (Carl Roth, Karlsruhe, Germany). Immunofluorescence staining was performed to evaluate the morphology of OCs and validate the method used for OC differentiation. Therefore, the cells were permeabilized using 0.1% Triton-X 100 (Sigma Aldrich, St. Louis, MO, USA) and 1% bovine serum albumin (BSA; Sigma Aldrich, St. Louis, MO, USA) was used as a blocking reagent. To categorize the cells into different stages of osteoclastogenesis, we used the following reagents: an OC-specific polyclonal anti-human Calcitonin Receptor (CT-R) antibody (Biozol, Eching, Germany) and a recombinant anti-TRAP (Tartrate-resistant acid phosphatase) antibody (Abcam, Cambridge, UK) to stain OC and OC precursors; the Phalloidin-iFluor 594 Reagent (Abcam, Cambridge, UK) for detecting actin filaments in the cytoskeleton; and DAPI (4’,6-Diamidino-2-Phenylindole, Thermo Fisher Scientific, Waltham, MA, USA) for staining cell nuclei. TRAP^+^ cells with less than three nuclei were considered as OC precursor cells, whereas cells that were TRAP^+^ CalcR^+^ with three or more nuclei were defined as mature OCs.

### TRAP staining

After verifying OC morphology, TRAP staining was performed on day 14 of OC differentiation to identify mature OCs and OC precursor cells. Therefore, the fixed cells were washed twice with PBS and incubated with TRAP buffer (pH 5; distilled water containing 6.56 g/L sodium acetate, AppliChem, Darmstadt, Germany; 23 g/L sodium tartrate dihydrate, Carl Roth, Karlsruhe, Germany) for 10 minutes. After another wash step, the cells were incubated with the TRAP staining solution (distilled water containing 0.1 g/L Naphtol aminoacid-MX phosphate, AppliChem, Darmstadt, Germany; 10 mL/L N,N-Dimethylformamide, AppliChem, Darmstadt, Germany; 0.6 g/L Fast Red Violet LB salt (Sigma-Aldrich, St. Louis, MO, USA) for 2 hours. After a final washing step with PBS, cover slides were applied.

### Quantitative and qualitative analysis of osteoclastogenesis

For the extensive evaluation of osteoclastogenesis on application of various checkpoint modulators (proteins, antibodies, and small molecule inhibitors), we utilized the Olympus microscope IX81 and cellSens Dimension software (Olympus, Shinjuku, Tokyo, Japan). Pictures were taken at 4x magnification for whole-well analysis and at 10x magnification for detailed assessments. Images taken at different magnifications were used to assess the degree of OC differentiation for each treatment condition and cell donor.

Using the cellSens Dimension software, the average size of TRAP^+^ cells was analyzed in six squares (each measuring 1 mm^2^) by automatic quantification of the area taken up by TRAP^+^ cells divided by the number of TRAP^+^ cells. This method was used for the initial examination of OC differentiation as mature OCs are typically larger than their precursor cells. Further, the number of all TRAP^+^ cells was counted manually in the same six squares and classified into precursor cells (< 3 nuclei), small (3 – 10 nuclei) or giant (> 10 nuclei) OCs. In addition, the total number of OCs (> 3 nuclei) per square was assessed. Moreover, the percentage change in the TRAP^+^ cell area as well as the OC cell number was evaluated between different treatment conditions and compared to the control group. Lastly, the percentage of giant OCs was calculated to examine whether a specific OC population was predominantly responsible for causing changes in the total OC number.

### OC lysis activity

For generation of functional data, PBMCs were isolated as described above and the cell pellet was resuspended in MEMα containing 20% heat-inactivated FBS, 2 g/L HEPES, 1% L-glutamine, 1% PS and 30 ng/mL M-CSF for pre-differentiation. After 7 days, the OC precursors were trypsinized and seeded into 96-well plates at 30,000 cells per well. Based on a protocol by Tas and Bhaduri (34), the wells were coated with bonelike apatitic calcium phosphate. Differentiation of mature OCs was started by adding 30 ng/mL M-CSF and 60 ng/mL RANKL to the standard medium. The reagents indicated in **Supplementary Table 1** were added to the differentiation medium for investigation of their effects on OC activity. Differentiation medium was changed every 48 – 72 h.

After 9 days, von Kossa staining was performed to stain the remaining bonelike coating for analysis of the OC lysis activity. Therefore, the cells were treated with a 1M sodium hypochlorite solution (Carl Roth, Karlsruhe, Germany) and washed twice with PBS, followed by incubation with 5% AgNO_3_ (Carl Roth, Karlsruhe, Germany). After washing, sodium carbonate formaldehyde solution (Carl Roth, Karlsruhe, Germany) was applied, followed by washing with water and neutralization with 5% sodium thiosulfate (Carl Roth, Karlsruhe, Germany). After a final washing step, 100% ethanol (Carl Roth, Karlsruhe, Germany) was utilized for dehydration.

Pictures of the wells were taken at 10x magnification using the Olympus microscope IX81 and cellSens Dimension software. The remaining bonelike coating was analyzed in seven squares (each measuring 1mm^2^) by automatic area quantification and the actual OC resorption then calculated.

### Statistical analysis

All data were processed using Microsoft Excel (Microsoft Corporation, Redmond, WA, USA), and GraphPad Prism 9 (GraphPad Software, La Jolla, CA, USA) was utilized for statistical analysis. The D´Agostino-Pearson test or graphical analysis was performed to assess normality in all measured values. Statistical significance of the differences among all groups was evaluated using a one-way ANOVA. The level of significance was set at *P* < 0.05 (* < 0.05, ** < 0.01, *** < 0.001, **** < 0.0001).

Multiple analysis squares were assessed per well and examined as individual results due to substantial heterogeneity in OC culture, not merely in between experiments and donors but also in single wells. Considering these results as independent findings rather than technical replicates respects the dispersion of data and ensures exact statistical examinations, as published elsewhere (35, 36).

## Results

### Systematic approach to OC quantification and structured analysis

**Figure 1:**
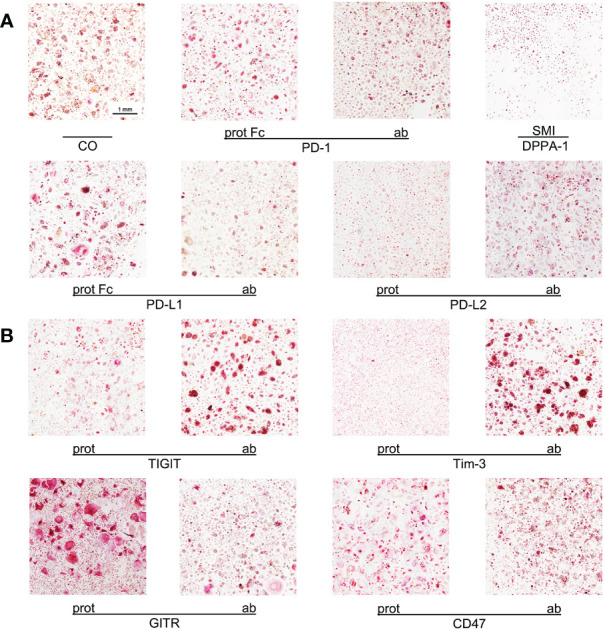
Imaging of OC differentiation under different treatment conditions. Representative images (captured at 10x magnification) of OC differentiation on day 14 after induction with different checkpoint modulators. **(A)** Group of PD-1-related checkpoint molecules. **(B)** Group of checkpoint molecules that are not yet approved for clinical use. CO, control group; prot, protein; ab, antagonistic antibody; PD-1, programmed cell death protein 1; SMI, small molecule inhibitor; PD-L1, programmed cell death 1 ligand 1; PD-L2, programmed cell death 1 ligand 2; TIGIT, T-cell immunoreceptor with immunoglobulin and ITIM domains; Tim-3, T-cell immunoglobulin and mucin-domain containing-3; GITR, glucocorticoid-induced TNFR-related protein; CD, cluster of differentiation.

In this study, we analyzed the effects of selected immune checkpoint proteins and their corresponding antibodies on osteoclast biology. The exact methodical approach consisted of OC induction from isolated PBMCs, followed by treatment with checkpoint-targeting reagents. On day 14, cells were fixed and analyzed for the expression of TRAP (**Supplementary Figure 2A**; **Figure 1**; **Supplementary Figure 5**). Immunofluorescence staining was subsequently performed to evaluate OC and OC precursor morphology (**Supplementary Figure 2B**). Such verification was essential as TRAP^+^ cells have a remarkable degree of heterogeneity in form, size, and the number of nuclei.

To generate highly reliable and representative data, we analyzed multiple complete wells, containing cells from different donors, in various experiments. First, images of culture wells, captured at a lower magnification, were used to provide a general overview. Next, detailed examinations were performed manually using images taken at a higher magnification. Although this manual approach was rather time-consuming, it generated precise results as numerous single values were surveyed and enabled an accurate analysis of various different treatment conditions. This approach respects the statistically relevant data distribution that can not only be detected in between experiments and donors but especially in each well of OC culture, hence, the analysis squares are examined as independent findings to further avoid study bias.

### Average size of TRAP^+^ cells

At first, the average surface taken up by a TRAP^+^ cell following induction was analyzed. We therefore measured the area taken up by all TRAP^+^ cells in multiple squares per culture well and divided this value by the cell number. The underlying idea was that the size of such cells—either OCs or OC precursors—correlates with the degree of OC differentiation, because OCs tend to be bigger than OC precursors. Also, OC size does not differ significantly and shows similar variability between all applied treatments tested in this study. Hence, surface area examination enables us to gain an overview of variations in TRAP^+^ cell size caused by specific checkpoint inductions, that could indicate how cells during OC differentiation were affected in comparison to the control and also to the corresponding protein or antibody.

**Figure 2:**
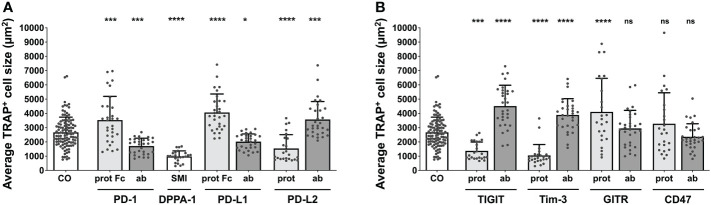
Assessment of average TRAP^+^ cell size under different treatment conditions. Average size of TRAP^+^ cells calculated in the six squares (each measuring 1 mm^2^) per culture well. **(A)** Group of PD-1-related checkpoint molecules. **(B)** Group of checkpoint molecules that are not yet approved for clinical use. Each data point represents the mean TRAP^+^ cell size, analyzed in a square of 1 mm^2^, of which 6 were assessed per culture well. Columns show mean and standard deviation. Asterisks indicate level of significance in comparison to CO, set at *P* < 0.05 (* < 0.05, *** < 0.001, **** < 0.0001; ns, non-significant). All data is acquired from at least 3 independent experiments, each with a minimum of 2 donors.

The average size of a TRAP^+^ cell in the control group was 2669 µm^2^. All comparisons for the group of PD-1 related checkpoints (**Figure 2A**) were significant. The PD-1 protein, PD-L1 protein, and PD-L2 antibody all caused an increase in cell size, with the largest size being attributed to OCs stimulated with the PD-L1 protein (4064 µm^2^ per cell). Treatment with the PD-1 protein and the PD-L2 antibody resulted in almost equal average cell sizes of 3532 µm^2^ and 3573 µm^2^, respectively. A decrease in the average cell size of TRAP^+^ cells could be observed following treatment with the PD-1 antibody, PD-L1 antibody, PD-L2 protein, and DPPA-1, the latter of which was associated with the greatest cell size reduction (981 µm^2^). For nivolumab, a significant decrease in the average TRAP^+^ cell size was detected at concentrations of 10 µg/mL (1603 µm^2^) and 100 µg/mL (1446 µm^2^) (**Supplementary Figure 6A**).

Concentrating on the remaining molecules (**Figure 2B**), the greatest increase in the cell size of TRAP^+^ cells to 4516 µm^2^ was registered for TIGIT antibody, followed by GITR protein (4106 µm^2^) and Tim-3 antibody stimulation (3894 µm^2^). In contrast, a significant decrease in average cell size was seen for the TIGIT protein (1369 µm^2^) and the Tim-3 protein (1057 µm^2^). Treatment with the GITR and CD47 antibodies as well as the CD47 protein did not lead to any significant changes in cell size (2947 µm^2^, 2357 µm^2^ and 3278 µm^2^, respectively).

These initial results highlighted the influence of various checkpoint molecules on OC differentiation. Interestingly, treatment with checkpoint proteins induced opposing outcomes to that of their corresponding antibodies, which was also appliable for the reagent DPPA-1, as an antagonist of both PD-1 and PD-L1.

### Percentage changes in the average size of TRAP^+^ cells

To further develop our analysis of the average surface area taken up by a TRAP^+^ cell under tissue culture conditions, percentage changes were calculated to determine the impact of various checkpoint modulators on osteoclastogenesis. Such relative changes are utilized for ease of comparability, as OCs are generally bigger than their precursors, allowing the assumption that an increase in cell size correlates with TRAP^+^ cell number.

With the size change for control inductions at 1.46%, augmentation of osteoclastogenesis was represented by higher percentual values. For the PD-1-associated group (**Supplementary Figure 3A**), the strongest effect was measured for the PD-L1 protein (+95.36%), followed by the PD-1 protein with an increase of +68.2%. A negative effect on cell size was induced by DPPA-1 (-50.83%) as well as the PD-L2 protein (-31.85%). PD-1, PD-L1 and PD-L2 antibodies led to non-significant findings (-17.72%, -2.83%, 14.05%, respectively). A considerable decrease in cell size was also caused by nivolumab when used at 10 µg/mL (-34.95%) and 100 µg/mL (-38.9%; **Supplementary Figure 6B**).

For the second checkpoint modulator group (**Supplementary Figure 3B**), the highest positive percentual change was seen for the GITR protein, which almost doubled the TRAP^+^ cell size (+95.63%), followed by the CD47 protein and the TIGIT antibody. In contrast, the maximal negative effect was caused by Tim-3 protein stimulation (-44.33%). Non-significant changes could be seen for treatment with the TIGIT protein and the Tim-3, GITR and CD47 antibodies.

### Quantifying total OCs

**Figure 3:**
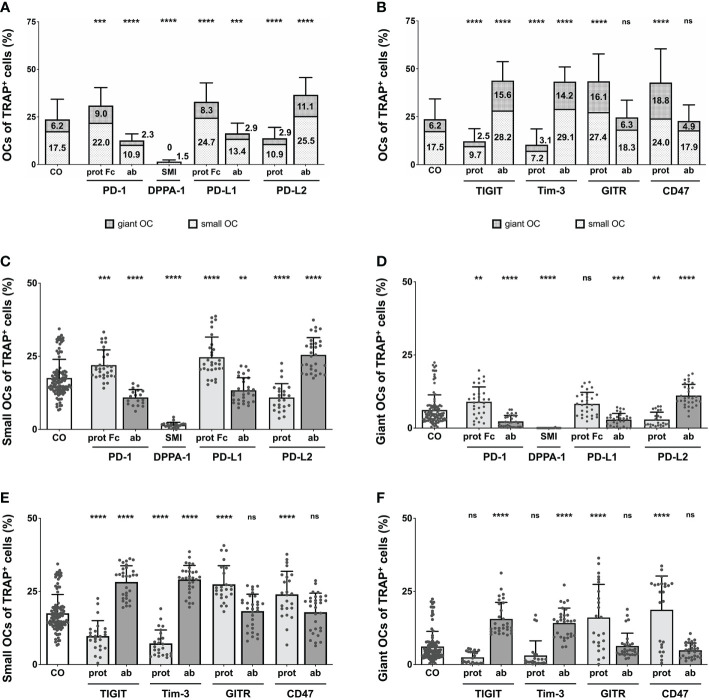
Analysis of the proportions of OCs of TRAP^+^ cells under various treatment condition. **(A, B)** OCs of TRAP^+^ cells as counted in the six squares (each measuring 1 mm^2^) per culture well. Columns are made up of the proportions of small OCs (TRAP^+^, 3 – 10 nuclei) and giant OCs (TRAP^+^, >10 nuclei) and labelled with the percentual values, respectively. Significance and standard deviation bars refer to the proportion of total OCs. **(C, E)** Small OCs and **(D, F)** giant OCs were counted separately in each square. Significance and standard deviation bars refer to proportions of small and giant OCs, respectively. Each data point represents the percentage of **(A, B)** all, **(C, E)** small, **(D, F)** giant OCs of TRAP^+^ cells, analyzed in a square of 1mm^2^, of which 6 were assessed per culture well. Columns show mean and standard deviation. Asterisks indicate level of significance in comparison to CO, set at *P* < 0.05 (** < 0.01, *** < 0.001, **** < 0.0001; ns, non-significant). All data is acquired from at least 3 independent experiments, each with a minimum of 2 donors.

In an effort to validate our previous analyses, the total number of OCs was counted manually for each treatment condition. Due to high variability in total cell number per induction well, we chose to utilize the percentage of osteoclasts within the total TRAP^+^ cell population. In the PD-1-related induction conditions (**Figure 3A**), each effect was highly significant (reaching levels of *P* < 0.0001), compared to the control (23.71% OCs). The highest number of OCs (36.61%) was seen for the PD-L2 antibody, followed by the PD-L1 protein (32.96%), and the PD-1 protein (30.97%). Checkpoint inductions causing very similar reductions in OC number were the PD-1 antibody (12.66%), the PD-L1 antibody (16.28%), and the PD-L2 protein (13.83%). The absolute lowest OC number was measured for the DPPA-1 condition (1.53%). Reduced OC numbers were also found in the wells treated with nivolumab at 10 µg/mL (9.9%) and 100 µg/mL (5.7%), both highly significant in comparison to the control group (**Supplementary Figure 7A**).

Remarkably significant changes in OC number were also seen in wells treated with the second checkpoint modulator group (**Figure 3B**), most reaching very high levels of significances (*P* < 0.0001). Inductions causing an increase in absolute OC numbers were the TIGIT and Tim-3 antibodies as well as the GITR and CD47 proteins. GITR protein induction had the most positive effect on OC number (43.51% OCs), although treatment with the TIGIT antibody (43.83%), the Tim-3 antibody (43.31%) and the CD47 protein (42.73%), resulted in very similar values. Reduction in OC number was noticed following stimulation with the Tim-3 protein (10.35% OCs) or the TIGIT protein (12.2%). Very subtle, non-significant effects were recognizable for the GITR and CD47 antibody inductions (24.63% and 22.75% OCs, respectively).

Overall, the effect of each checkpoint molecule on osteoclastogenesis could be confirmed by changes in OC number. Treatment of cells with the GITR and CD47 antibodies did not significantly change OC number compared to the control, although, they did lower OC numbers considerably in relation to their corresponding proteins.

### Percentage changes in total OC number

Continuing our analysis of total OC numbers, percentage changes were determined for ease of comparison to the control group (+0.15%) and assessment of checkpoint-specific effects on osteoclastogenesis. Higher percentual changes represented an increase in osteoclastogenesis. Among the PD-1-related inductions, the strongest effect was recognized for PD-L1 (+53.43%; **Supplementary Figure 4A**). A decrease in OC number was caused by the PD-L2 protein, the PD-1 and PD-L1 antibodies. As an inhibitor of the PD-1/PD-L1 interaction, DPPA-1 exhibited the highest overall reduction in OCs (-91.7%). The PD-1 antibody and the PD-L2 protein induced a rather moderate decrease in OC number (-39.51% and -40.28%, respectively). Moreover, a negative percentual change was caused by nivolumab at 10 and 100 µg/mL (-49.59% and -69.81%, respectively; **Supplementary Figure 7B**).

In the second checkpoint group (**Supplementary Figure 4B**), an increase in total OC numbers was caused by the TIGIT and Tim-3 antibodies, and the GITR and CD47 proteins (all resulted in an increase of at least +50%). The GITR protein was responsible for the most marked increase in total OC number (+67.99%). In contrast, the Tim-3 and TIGIT proteins reduced OC numbers (-61.59% and -51.98%, respectively). Non-significant reduction could be seen for the GITR and CD47 antibodies.

### Lysis of bonelike coating

Further investigating these findings, the influence of both checkpoint groups on the functional activity of OCs was examined and lysis of a bonelike apatitic calcium phosphate coating (**Supplementary Figure 8A**) was quantified. Compared to the control group at 46.57%, higher percentual values symbolize increased functional activity of OCs. In the PD-1-related checkpoint group (**Supplementary Figure 8B**), the significantly highest OC activity could be found for PD-L1 protein (90.23%), whereas significantly reduced resorption was caused by the PD-1 antibody (20%) and the lowest lytic activity could be seen for DPPA-1 at only 0.04%. Non-significant effects were detected after application of PD-1 and PD-L2 proteins, PD-L1 antibody as well as the PD-L2 antibody, the latter presenting a non-statistically significant trend at 70.81% lysed coating.

For the second checkpoint group (**Supplementary Figure 8C**), TIGIT and Tim-3 antibodies increased the OC activity significantly (76.85% and 75.89%, respectively). In contrast, GITR antibody diminished the lysis activity significantly (18.41%). All applied checkpoint proteins and the CD47 antibody induced non-significant findings.

### Quantifying small and giant OCs

To validate our previous results, we counted the number of small and giant OCs in our cultures. In this study, small OCs were considered as TRAP^+^ cells with 3 – 10 nuclei, whereas giant OCs were TRAP^+^ and had 10 or more nuclei per cell. This separation between small and giant OCs was intended to determine whether one of these OC subtypes was responsible for the changes in total OC number.

For the control group, the level of small OCs was at 17.54%, whereas giant OCs made up 6.17% of the TRAP^+^ cells. Focusing on PD-1-related inductions (**Figures 3C, D**), the overall trend seen for total OC numbers was very similar for the numbers of small and giant OCs. Examining small OCs (**Figure 3C**), the PD-L2 antibody induced the largest number of small OCs (25.49% of TRAP^+^ cells), closely followed by the PD-L1 protein (24.67%) and the PD-1 protein. A negative effect on the number of small OCs was detected for the PD-1 and PD-L1 antibodies, the PD-L2 protein, and DPPA-1 (1.51% small OCs). Regarding giant OCs (**Figure 3D**), the effects were equal for each induction compared to the numbers of small OCs. The maximal number of small OCs was induced by the PD-L2 antibody (11.12%), followed by the PD-1 protein (8.99%), whereas the minimal number was observed for DPPA-1 (0.01%). Further significant reduction (compared to the control) could be seen for the PD-1 and PD-L1 antibodies (2.27% and 2.88%, respectively) as well as the PD-L2 protein (2.92%). Small OCs were significantly reduced following treatment with 10 and 100 µg/mL nivolumab (8.21% and 4.5%, respectively; **Supplementary Figure 7C**). In addition, a highly significant decrease in giant OCs was detectable at 10 and 100 µg/mL nivolumab (1.66% and 1.22%, respectively; **Supplementary Figure 7D**).

Concentrating on the second group of OC inductions (**Figure 3E, F**), the previously recognized trend was also mostly followed. Positive effects on the number of small OCs (**Figure 3E**) were seen for stimulation with the TIGIT and Tim-3 antibodies, as well as the GITR and CD47 proteins. Maximal small OC numbers were seen for the Tim-3 antibody condition (29.12%) and minimal for the Tim-3 protein (7.24%). GITR and CD47 antibodies did not follow the overall trend as only slight, non-significant increases in the number of small OCs (18.29% and 17.89%, respectively) were registered. The TIGIT and Tim-3 antibodies, and the GITR and CD47 proteins were better at inducing giant OC numbers with the maximum number of giant OCs observed for CD47 protein condition (18.73%; **Figure 3F**). In contrast, TIGIT protein application showed a non-significant trend for reducing the giant OC number to 2.48%.

### Proportion of giant OCs within the total OC number

**Figure 4:**
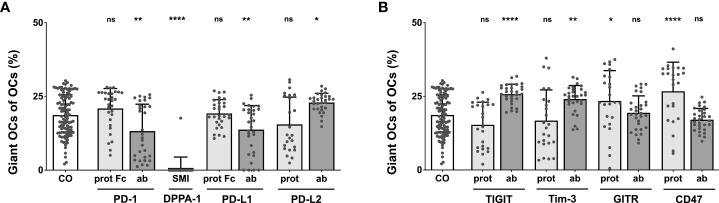
Analysis of the percentage of giant OCs that make up total OC numbers. Giant OCs make up a significant proportion of total OC. As all inductions are well below 50%, alterations in total OC number are mostly due to changes in the number of small OCs. **(A)** Group of PD-1-related checkpoint molecules. **(B)** Group of checkpoint molecules that are not yet approved for clinical use. Each data point represents the percentage of giant OCs of the total OC number, analyzed in a square of 1 mm^2^, of which 6 were assessed per culture well. Columns show mean and standard deviation. Asterisks indicate level of significance in comparison to CO, set at *P* < 0.05 (* < 0.05, ** < 0.01, **** < 0.0001; ns, non-significant). All data is acquired from at least 3 independent experiments, each with a minimum of 2 donors.

Completing the analysis of the effects exerted by checkpoint modulators on OC differentiation, the percentage of giant OCs within the total OC number was calculated. In the control group, giant OCs made up 18.69% of all OCs. Starting with the PD-1-related checkpoint modulators (**Figure 4A**), the highest proportion of giant OCs was seen in wells treated with the PD-L2 antibody (22.91%), which represented a significant increase compared to the control. The smallest composition of giant OCs was identified in the DPPA-1-treated wells (0.77%). Other conditions that showed a relevant reduction in the proportion of giant OCs were the PD-L1 antibody (13.76%) and the PD-1 antibody (13.24%). Treatment with 10 µg/mL nivolumab resulted in significantly lower percentages of giant OCs (12.33%; **Supplementary Figure 9**). Insignificant changes were found for the PD-1, PD-L1 and PD-L2 proteins (20.9%, 19.25% and 15.52%, respectively).

Focusing on the second checkpoint group (**Figure 4B**), maximal giant OC percentages were elicited by the CD47 protein (26.74%), followed closely by the TIGIT antibody (25.77%), the Tim-3 antibody (24.1%), and the GITR protein (23.44%); all these changes were statistically significant. The proportion of giant OCs did not result in statistically relevant changes relative to the control when induced with the TIGIT protein (15.35%), the Tim-3 protein (16.79%), or the GITR (19.41%) and CD47 antibodies (17.07%).

Generally, both subgroups of OCs (small vs. giant) showed similar perturbations in number under most conditions, as could have already been assumed from prior analyses. It became further apparent that for every induction condition tested, giant OCs made up the unmistakable minority of all OCs. Therefore, changes in the total number of OCs were primarily caused by alterations in the quantity of small OCs.

**Figure 5:**
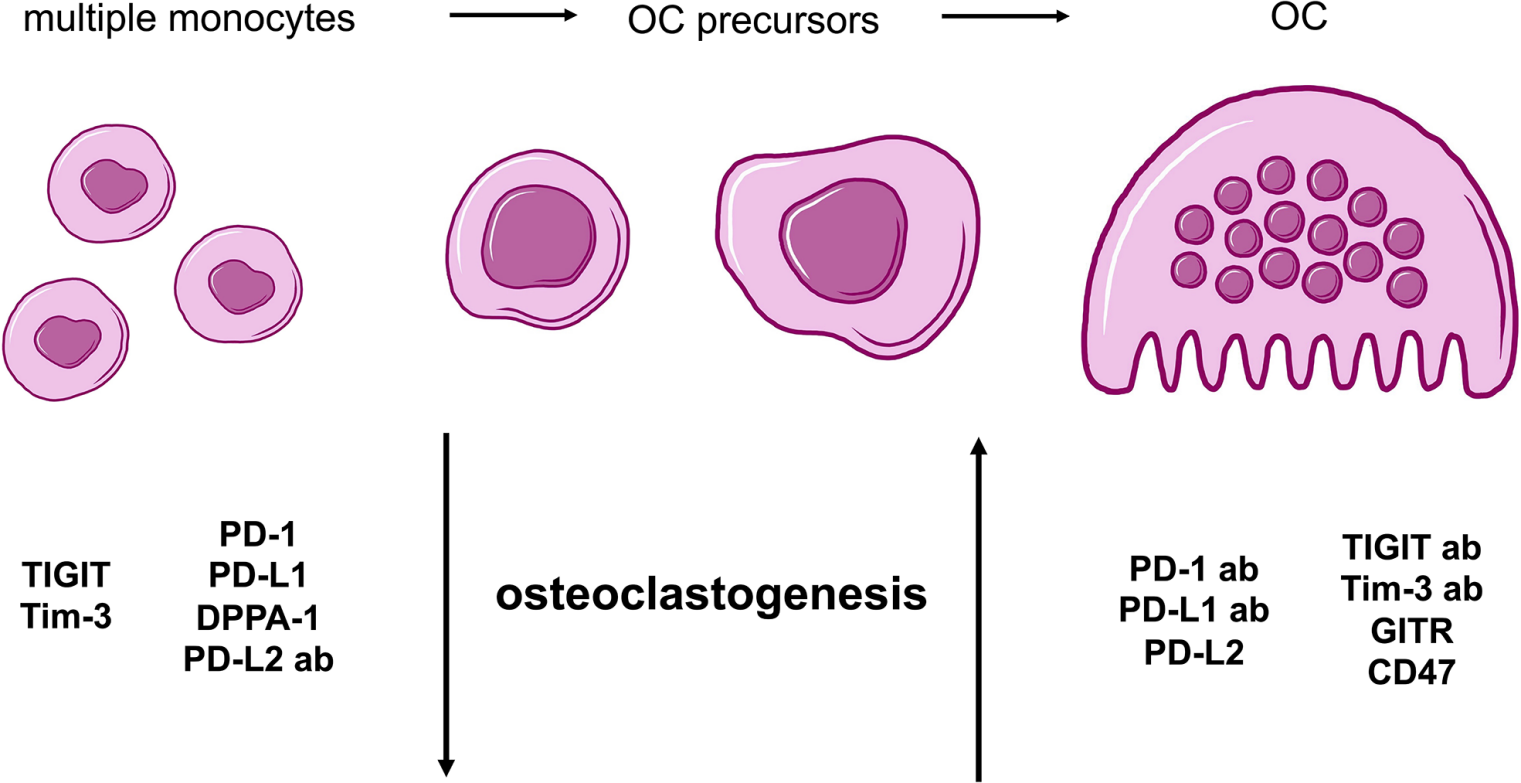
Summarizing cartoon of effects caused by agonistic and antagonistic targeting of immune checkpoint molecules during osteoclastogenesis. OC differentiation begins with multiple PBMCs that evolve into OC precursors and finally, mature OCs. For our study, two general groups of checkpoints can be distinguished based on characteristic alterations of osteoclastogenesis. Molecules with negative effect on osteoclastogenesis and therefore, a higher number of monocytes and OC precursors, are TIGIT, Tim-3, PD-1, PD-L1 proteins as well as DPPA-1 and PD-L2 antibody. In contrast, PD-1, PD-L1, TIGIT and Tim-3 antibodies as well as PD-L2, GITR and CD47 proteins present stimulation of OC development.

In conclusion, all analyses that were performed to investigate the effects of checkpoint proteins and their antagonistic antibodies on osteoclast biology showed consistent results. The combination of data obtained from the comparison of average TRAP^+^ cell sizes and functional OC activity as well as the analysis of OC numbers, demonstrated that osteoclastogenesis is sensitive to checkpoint modulation. Furthermore, we are able to categorize our findings into two overarching groups: 1) Checkpoint modulators that had a positive effect on osteoclastogenesis, namely, PD-1 and PD-L1 proteins, the PD-L2 antibody, the TIGIT and Tim-3 antibodies, as well as the GITR and CD47 proteins; and 2) Corresponding antibodies or checkpoint proteins that caused negative effects on osteoclastogenesis, namely, the PD-1 and PD-L1 antibodies, DPPA-1, the PD-L2, TIGIT, and Tim-3 proteins, as well as the GITR and CD47 antibodies (**Figure 5**).

## Discussion

Results obtained in this study enabled the categorization of checkpoint modulators in two groups that influence osteoclastogenesis differently. In this regard, we found PD-1, PD-L1, GITR, and CD47 proteins to be OC stimulatory, whereas, PD-L2, DPPA-1, TIGIT, and Tim-3 showed inhibitory effects on osteoclastogenesis. The corresponding antagonistic antibodies caused contrary results (**Figure 5**).

### PD-1

Our examinations showed an increase in osteoclastogenesis and in the size of OCs and OC precursors after application of the PD-1 protein. It is widely accepted to compare bone resorption by OCs (as professional phagocytes) to macrophage phagocytosis during pro-inflammatory M1 polarization (37–40). Such consideration is even more reasonable owing to PD-1 being significantly upregulated in PBMCs of patients suffering from postmenopausal osteoporosis, which represents an imbalance in the OC/OB activity ratio and further correlates with elevated C-reactive protein (CRP) levels (2). Hence, PD-1 is thought to favor OC maturation not only *via* the inhibition of osteoblastic bone formation but also by upregulating OC activity as well as inflammatory mediators (2). Moreover, overactive OCs have been detected following PD-1 pathway stimulation (41). Thus, raised PD-1 levels are thought to represent ongoing inflammatory immunity, which favors bone degradation and clinically correlates with osteoporosis (42). However, inhibition of PD-1 signaling in a murine bone cancer model resulted in less bone destruction (43), leading to the assumption that overstimulating the PD-1 pathway might lead to the development of an osteoporotic bone condition due to OC activation. As could be expected, administration of an inhibitory PD-1 antibody led to significantly reduced levels of OC formation and size, in our study. Findings of osteopetrosis in mice after PD-1-ICI and genetic knockout (2, 44) support our results of impaired OC activation after PD-1 blockade.

Controversially, there is some evidence of PD-1 deficiency causing osteoporosis. A higher risk of fracture was reported after anti-PD-1 therapy (1). However, owing to only six patients being included in this particular study, findings may have been coincidental. A reduction in trabecular bone volume and evidence of a weakened microstructure due to increased RANKL production were described in a murine model (42). However, the study’s authors ultimately agreed on the essentiality of the PD-1 signaling pathway for regulating osteoclastogenesis and maintaining optimal bone structure and density.

### PD-L1

PD-L1, as a ligand of PD-1, participates in the PD-1 pathway, which is immensely important for bone homeostasis. Similarly to PD-1, we found that induction with PD-L1 increased OC size and number *in vitro*, albeit, to a lesser extent than its receptor. In contrast, the PD-L1 antibody reduced OC formation, again less strongly than the PD-1 antibody. Considering that OC activation might be comparable to the activation of immune cells, published results are not in agreement with our findings, as PD-L1 T cells promote transition to the immunosuppressive M2 profile similar to what is seen regarding PD-1 and its influence on macrophages (45). In line with these results, anti-PD-L1 therapy is reported to favor the pro-inflammatory M1 macrophage phenotype (46, 47). However, these findings were obtained using T cells and macrophages, which due to particular dissimilarities with OCs, are not readily applicable to our study design.

In our study, the effect of the inhibitory PD-L1 antibody on osteoclastogenesis was reproduced by application of nivolumab, another anti-PD-L1 modulator. It even became apparent that the blockade of osteoclastogenesis caused by nivolumab was dose-dependent, ultimately suggesting that PD-L1 application indeed favors osteoclastogenesis whereas the inhibition of PD-L1 results in a reduction in OC size and number.

### PD-L2

In contrast to PD-1 and PD-L1, PD-L2 reduced the quantity of newly formed OCs whereas the corresponding antagonistic antibody increased osteoclastogenesis. Hence, the actions of this second PD-1 ligand highlight the alternate functions of the PD-1 pathway. These findings are in line with a study published by Greisen et al., who found that PD-L2 inhibited TRAP activity in RANKL- and MCSF- stimulated cultures as well as reduced the activity and development of ACPA-depending OCs on synthetic calcium phosphate-coated plates, while not having any effect on OBs (48). Likewise, mice deficient in PD-L2 were associated with diminished bone density and impairments in bone microstructure (48), thus supporting our *in vitro* results.

### PD-1-associated molecules

Comparing all the modulators of the PD-1 pathway examined in this study, it becomes apparent that the antagonistic PD-1 antibody showed a stronger negative influence on OCs than the PD-L1 antibody, whereas the proteins presented caused contrary effects. The inhibitory effect of PD-L2 could have been expected to dominate when only PD-L1 was blocked and therefore, osteoclastogenesis to be even more restricted compared to PD-1 blockade. As this was not the case, it seems plausible that other ligands or co-factors influence the regulation of OCs by the PD-1 family. Reciprocal interactions of PD-1 and its ligands can also be imagined, as these might be interrupted by PD-L1 blockade and thus, inhibitory effects were not as strong as could have been anticipated.

Reciprocal effects generated by the PD-1 ligands can be explained by a multitude of different factors. First, PD-L1 is widely expressed on hematopoietic and non-hematopoietic cells, whereas expression of PD-L2 is restricted to APCs, as, e.g., dendritic cells, macrophages, monocytes and some B cells (49, 50). It is unclear whether, and if so, how, this influences effects on OCs (51), however, it can be imagined that PD-L1 improves osteoclastogenesis in early phases in contrast to PD-L2 that might alter OC generation in later stages. Further, both ligands compete for binding and present majorly different molecular interaction with PD-1 (52). PD-L1 shows poor affinity to PD-1 due to rapid biphasic dissociation and lack of conformation change for efficient interaction (52). PD-L2 however fits much better to PD-1 and binds 3x stronger, this is attributed to a PD-L2-specific “latch” supposed to improve PD-1-PD-L2 binding (49) as well as to modification of the binding cavity in PD-1 after PD-L2 binding (50). Also, PD-L2 presents constant dissociation from PD-1 and could therefore outcompete PD-L1 effects (51, 52), resulting in inhibition of osteoclastogenesis. Be that as it may, both PD-1 ligands are able to hinder binding of the other ligand dose-dependently (49, 52), hence, this topic should be researched further.

Despite this, the inhibitory effects of PD-L1 inhibition were confirmed using nivolumab, which resulted in a similar reduction in osteoclastogenesis, as reflected by lower OC number and size. Moreover, DPPA-1, as a potent inhibitor of the PD-1/PD-L1 interaction showed the strongest negative effect on OC maturation and thereby verified previous findings.

### TIGIT

TIGIT protein application resulted in a reduction in OC size and number, consistent with findings generated from studies of macrophages and monocytes. TIGIT is necessary for regulation of these mononuclear cells (53) insofar as it downregulates immune activity, reduces activation of mouse peritoneal macrophages, and stimulates polarization towards the anti-inflammatory M2 phenotype *via* the induction of IL-10 expression (54).

In our study, TIGIT antibody induction showed contrary effects and significantly promoted OC formation. In accordance, Chen et al. demonstrated that TIGIT knockout mice exhibited an M1 macrophage phenotype and were therefore more susceptible to autoimmune diseases (54).

Regarding calcium phosphate lysis, TIGIT protein treatment elevated the activity, contrary to the quantitative examinations. Still, the overall tendency of TIGIT regulation is sustained as, in comparison, TIGIT antibody application resulted in considerably raised lysis of bonelike coating.

### Tim-3

Similar to TIGIT, Tim-3 protein induction resulted in impaired osteoclastogenesis, in agreement with published data; Tim-3 has been shown to limit the proportion of M1 macrophages by reducing pro-inflammatory molecules such as TNFα, IFN-γ, and IL-12 (53, 55). Similarly, its ligand galectin-9 is also known as a potent inhibitor of OC differentiation, which it does by reducing the levels of pro-inflammatory mediators such as IL-12, IL-17, and IFN-γ, as has been demonstrated in an *in vitro* culture of murine bone marrow macrophages (41, 56).

The antagonistic Tim-3 antibody used in our study increased the extent of osteoclastogenesis, correlating with a recent study that characterized anti-Tim-3 antibodies as pro-inflammatory due to an increase in IL-12, IL-6, and IL-10 levels being observed after antibody application. Of these, IL-12 acts as a potent inflammatory mediator that would likely increase OC numbers and favor bone destruction (55). Despite these findings, there is some evidence for the contrary effects of Tim-3. Moriyama et al. published that Tim-3 expression correlated with the proportion of TRAP^+^ cells as mononuclear precursors of OCs (56). Moreover, application of an inhibitory Tim-3 antibody reduced PD-1 expression on THP-1 cells, which is a cell line very similar to OCs (55). Considering our results, PD-1 deficiency would result in limited osteoclastogenesis in contrast to the increased OC levels caused by Tim-3 blockade. It was recently reported that PD-1 induction reduced OC numbers (42), which in this case would be analogous to our Tim-3 antibody data that favor osteoclastogenesis. Nevertheless, reduced PD-1 levels would probably affect the stimulation of OC development caused by an antagonistic Tim-3 antibody; however, it can be assumed that the Tim-3-related effect is predominant in terms of OC regulation. Nonetheless, comparing such intricate connections in response to Tim-3 application during osteoclastogenesis is rather difficult in THP-1 cells and therefore, these finding may not be readily transferable between systems.

### GITR

OC induction with the GITR protein resulted in an overall increase in OC size and number, which is consistent with current literature. *Via* interaction with its ligand GITRL, GITR stimulates osteoclastogenesis by stimulation of RANKL and downregulation of OPG, resulting in affected bone formation (57). Further, GITR plays a proinflammatory role in autoimmune diseases and chronic inflammatory conditions (58, 59). In this regard, GITR expression is also associated with the proinflammatory M1 macrophage phenotype and stimulation of inflammatory cytokines (57, 59), supporting our findings in OCs. Therefore, it seems reasonable to consider that GITR stimulation would reverse immunosuppression during periods of chronic inflammation, rendering GITR ICIs useful in the treatment of autoimmune disease.

In line with the previously discussed checkpoint modulators, it could be assumed that an antagonistic GITR antibody would reduce osteoclastogenesis. However, in our study the OC size and number remained similar to that of the control group, following GITR antibody treatment. This could possibly be explained by only minimal GITR protein expression in the culture medium that would have been antagonized by such a blocking antibody, resulting in only marginal alterations in OC size and count. Nevertheless, a slightly negative effect on osteoclastogenesis was recognizable for the GITR antibody during our analysis of percentage change in OC number data. A recent publication strengthens the assumption that an antagonistic GITR antibody would reduce OC numbers, as GITR deficiency was shown to decrease the proliferation and phagocytic ability of macrophages (59).

### CD47

Similarly to GITR, CD47 protein application resulted in stimulation of osteoclastogenesis. The interaction between CD47 and its ligand SIRPα presents a “don´t eat me” signal, which ultimately prevents phagocytosis by macrophages and OCs (60–63). Similar to the findings of Hobolt-Pedersen et al., who showed CD47^+^ OCs and precursor cells to be rather small compared to the often larger CD47^–^ OCs (60), the size of TRAP^+^ OCs and precursors generated as a result of CD47 application in our study, was the smallest compared to OCs cultured under all other experimental conditions.

We hypothesized that an inhibitory CD47 antibody would impact OC number. However, in this study, the number of OC remained similar to that of the control group (and the antagonistic GITR antibody) on application of anti-CD47. Again, we did not see significant changes in OC size after induction with the inhibitory CD47 antibody, in accordance with previous publications, in which CD47-deficient mice showed reduced OC surface area (61) and increased bone volume (64). Furthermore, significantly fever OCs were formed from CD47-deficient macrophages (64), likely due to the same mechanisms that are responsible for causing the breakdown of OC fusion, after antagonistic CD47 antibody application (60).

### Technical challenges in examination of quantity and quality of OCs

Our experimental approach in this study was slightly different to research previously performed in the complex field of OC biology. Our aim was to perform precise and objective analyses despite the immense level of variation in morphology of OCs and OC precursors. To ensure these qualities, we focused on rather labor-intensive manual examination of the samples instead of error-prone automatic analyses owing to current technical limitations. Thereby, we were able to recognize that changes in OC number were mostly due to alterations in the number of small OCs. Because small OCs are still larger in size than OC precursor cells, the analysis of average TRAP^+^ cell size provided useful insights. We found that the proportion of giant OCs correlated with total OC numbers, resulting in higher numbers of giant OCs under OC-stimulating inductions in contrast to conditions that reduced total osteoclastogenesis. Consequently, these findings suggest that small OCs are responsible for the major changes affecting OC number, whereas giant OC numbers are modified to a lesser extent. This could be due to the process of osteoclastogenesis in which many small OCs undergo fusion into fewer giant OCs, hence, the imbalance in OC sizes and multinuclearity. Moreover, OCs stay longer in their small form and are easier affected by apoptosis once they become multinucleated giant OCs, leading to the conclusion that such an effect is probably not caused by altered formation of OCs.

As a result, we highly recommend the analysis of OCs in complete culture wells and under at least two different levels of magnification. Furthermore, we suggest performing manual quantifications of OC number and size in a reasonably large cohort to counterbalance statistical discrepancies. Counting small and giant OCs separately also provides more insight into immune checkpoint regulated osteoclastogenesis. Despite being somewhat time-consuming, such an approach avoids considerable bias, improves the accuracy of results, and ultimately allows for the comparability and reproducibility of findings.

Despite the variety of checkpoint modulators tested in our study, the validity of results is ensured even though protein-antibody-interaction is necessary for actual effects. Indeed, multiple studies demonstrate that PBMCs express the ligands of the examined checkpoint proteins (49, 58, 65–68). Nonetheless, it is noteworthy that multiple factors regulate expression of checkpoint molecules and ligands which need to be further researched.

In conclusion, these results allow for a better understanding of potential bone-related side effects in patients that receive immune checkpoint inhibition as therapy for malignant disease. It seems plausible that adverse events such as bone fractures represent highly efficient or rather ‘too effective’ therapeutic approaches, considering that some patients who showed a complete clinical response to their cancer therapy after treatment with ICIs suffered bone fractures (3). Hence, the main challenge in cancer therapy is to balance successful anti-malignant therapy while preventing serious, often bone-related, adverse effects. Further research is needed to assess which checkpoint molecules and more specifically, which ICI therapies result in bone fractures, in addition to examining whether drug dose reduction may be used to manage treatment intensity. Moreover, these findings contribute to improving the insufficient treatment options for conditions such as osteoporosis, septic and aseptic loosening of endoprostheses, bone cancers, as well as side effects of cancer therapy. ICI of checkpoint molecules that upregulate OC levels as well as treatment with agonistic antibodies for stimulation of immune checkpoints responsible for reducing the extent of osteoclastogenesis, may become promising therapy options for the treatment of such conditions of impaired bone homeostasis. However, it should be investigated whether, and if so, how, these specific OC-modulating immune checkpoints affect other cells implicated in bone homeostasis while remembering the other systemic effects of such therapeutic approaches.

Recent studies (69–71) showed correlations of size, number and activity of OCs. Regarding a possible causal connection of these qualities, lysis of bonelike calcium phosphate was carried out and generally substantiates the trends we have seen for OC size and quantity. Such a widely accepted assay for examination of essential pre-resorptive OC activity (70, 72–75) leads to the assumption that these parameters may be related to a certain extent.

Likewise, bone resorption assays display another established method for evaluation of OC function (76, 77). Considering the performed screening approach, these are slightly problematic due to difficult analysis, lack of reproducibility and thus, less comparability of results. In comparison to *in vitro* OC culture, even more severe heterogeneity and variability is seen on bone slices due to bone-specific characteristics and influence on OC biology (78–80). Improving the reliability and affected statistical significance of results requires particularly high numbers of replicates that are – by now – unfeasible for such a large variety of checkpoint conditions. Hence, a direct checkpoint-mediated effect on bone resorption cannot be taken for granted though is rather likely. Despite these complicated aspects, bone resorption assays should definitely be included in further research of selected, particularly promising immune checkpoint and their effects on OC activity.

The lack of consensus between our results and the literature can be explained by fundamental differences between OCs and its precursor cells, human and murine OCs in general, and study conditions in particular. As it is well known that the action of immune checkpoints is highly cell-specific (8, 13, 16, 19), furthermore, due to the lack of OC-specific checkpoint publications, and particularly relating to human OCs, current literature is not sufficient for drawing well-founded conclusion on this topic. For this reason, it would be beneficial to examine the effects of these checkpoint molecules on other cells of relevance in bone regulation.

Our study served to facilitate the methodological approach currently used to conduct osteoclast research, leading to the generation of more objective and reproducible results. Such standardization of experimental research performed on human OCs could enable more accurate comparisons between findings than are currently possible using standard approaches. In addition, we observed significant effects of checkpoint modulation on osteoclast biology, which would be directly applicable to advancing the treatment of osteopenic conditions, such as osteoporosis, the aseptic loosening of endoprostheses, bone cancer, and bone-related side effects of cancer therapy.

## Data availability statement

The raw data supporting the conclusions of this article will be made available by the authors, without undue reservation.

## Ethics statement

The studies involving human participants were reviewed and approved by the ethics committee of the University of Bonn, Germany. The patients/participants provided their written informed consent to participate in this study.

## Author contributions

All authors listed have made a substantial, direct, and intellectual contribution to the work, and approved it for publication.

## Supplementary material

Supplementary Figure 1 Nonspecific OC controls. OCs of TRAP^+^ cells as counted per culture well after application of unspecific irrelevant protein (BSA) and antibody (rat IgG1, goat IgG, mouse IgG1 and IgG2b) controls in comparison to regular OC control group, resulting in non-significant alterations. Each data point represents the percentage of all OCs of TRAP^+^ cells, analyzed in a square of 1 mm^2^, of which 6 were assessed per culture well. Columns show mean and standard deviation referring to the pooled proportion of total OCs from 2 donors. BSA, bovine serum albumin; Ig, Immunoglobulin.

Supplementary Figure 2 OC differentiation methodology. (A) Outline of the OC differentiation protocol. Briefly, after isolation of peripheral blood mononuclear cells, osteoclastogenesis was induced by application of RANKL, MCSF, Vitamin D, and a checkpoint modulator. On day 14, fixation, OC validation and TRAP staining of the cells were performed. PBMC, peripheral blood mononuclear cells; OC, osteoclast; TRAP, Tartrate-resistant acid phosphatase; RANKL, Receptor Activator of NF-κB ligand; MCSF, Macrophage colony-stimulating factor; Vitamin D, 1α,25-Dihydroxyvitamin D3; checkpoint, checkpoint modulator (protein, inhibitory antibody or small molecule inhibitor). (B) Pictures of immunofluorescence staining for validation of OC morphology. The following structures were stained for classification of the cells: nuclei (blue), actin filaments (red), TRAP (green), and calcitonine receptor (yellow). OC precursor cells are defined as TRAP^+^ cells with less than 3 nuclei, whereas TRAP^+^ and CalcR^+^ cells with three or more nuclei are considered Ocs. Representative image, showing half of a tissue culture well with six analysis squares, each measuring 1 mm^2^. Two side-by-side images taken at the same level of magnification to demonstrate the immense inhomogeneities in OC morphology, size, and intensity of TRAP staining.

Supplementary Figure 3 Assessment of percentual changes of average TRAP^+^ cell size under different treatment conditions. Percentage change in average TRAP^+^ cell size, compared to the control group. (A) Group of PD-1-related checkpoint molecules. (B) Group of checkpoint molecules that are not yet approved for clinical use. Each data point represents the percentual change of the average TRAP^+^ cell size in comparison to the mean control values, analyzed in a square of 1 mm^2^, of which 6 were assessed per culture well. Columns show mean and standard deviation. Asterisks indicate level of significance in comparison to CO, set at *P* < 0.05 (* < 0.05, ** < 0.01, *** < 0.001, **** < 0.0001). All data is acquired from at least 3 independent experiments, each with a minimum of 2 donors.

Supplementary Figure 4 Assessment of percentual changes of the total OC number under different treatment conditions. Percentage change in the proportion of total OCs, compared to the control group. (A) Group of PD-1-related checkpoint molecules. (B) Group of checkpoint molecules that are not yet approved for clinical use. Each data point represents the percentual change of the total OC number in comparison to the mean control values, analyzed in a square of 1 mm^2^, of which 6 were assessed per culture well. Columns show mean and standard deviation. Asterisks indicate level of significance in comparison to CO, set at *P* < 0.05 (* < 0.05, ** < 0.01, *** < 0.001, **** < 0.0001). All data is acquired from at least 3 independent experiments, each with a minimum of 2 donors.

Supplementary Figure 5 Imaging of OC differentiation under different treatment conditions. Representative images (captured at 10x magnification) of OC differentiation on day 14, following treatment with different checkpoint modulators. Nivo, nivolumab; 10, 10 µg/mL; 100, 100 µg/mL

Supplementary Figure 6 Analysis of average size of TRAP^+^ cells and percentual changes. (A) Average size of TRAP^+^ cells calculated in six squares (each measuring 1 mm^2^) per culture well. (B) Percentual change of average TRAP^+^ cell size compared to the control group. Each data point represents (A) the average size of TRAP^+^ cells and (B) the percentual change of the cell size, analyzed in squares of 1 mm^2^, of which 6 were assessed per culture well. Columns show mean and standard deviation. Asterisks indicate level of significance in comparison to CO, set at *P* < 0.05 (* < 0.05, ** < 0.01, *** < 0.001, **** < 0.0001). All data is acquired from at least 2 independent experiments, each with a minimum of 3 donors.

Supplementary Figure 7 Assessment of the percentage of OC numbers of TRAP^+^ cells and percentual changes. (A) OCs of TRAP^+^ cells as counted in the previously utilized six squares (each measuring 1 mm^2^) per culture well. Columns are divided by the percentage of small OCs (TRAP^+^, 3 – 10 nuclei) and giant OCs (TRAP^+^, >10 nuclei) and labelled with the percentual values, respectively. Significance and standard deviation bars refer to proportion of total OCs. (B) Percentual change in the total OC population, compared to the control group. (C) Small OCs and (D) giant OCs were counted separately in each square. Significance and standard deviation bars refer to proportions of small and giant OCs, respectively. Each data point represents (A) the percentage of the total OC number of TRAP^+^ cells, (B) its percentual change compared to the Control, and the percentage of (C) small and (D) giant OCs, analyzed in squares of 1 mm^2^, of which 6 were assessed per culture well. Columns show mean and standard deviation. Asterisks indicate level of significance in comparison to CO, set at *P* < 0.05 (* < 0.05, ** < 0.01, *** < 0.001, **** < 0.0001). All data is acquired from at least 2 independent experiments, each with a minimum of 3 donors.

Supplementary Figure 8 Lysis of bonelike apatitic calcium phosphate coating. For evaluation of OC functional activity, lysis of a calcium phosphate coating was evaluated after application of checkpoint regulators during osteoclastogenesis. (A) Comparison of exemplary pictures of von Kossa-stained bonelike coating (captured at 10x magnification) without any treatment (∅) and after application of OC differentiation medium for 9 days (CO). Remaining coating is stained black whereas white areas represent lysed calcium phosphate coating. Percentages of resorbed coating of (B) PD-1 related checkpoints and (C) immune checkpoint molecules of the group of checkpoint modulators that are not yet clinically used were analyzed in 7 squares per culture well, each measuring 1mm^2^. Columns show mean and standard deviation. Asterisks indicate level of significance in comparison to CO, set at *P* < 0.05 (* < 0.05, ** < 0.01, *** < 0.001, **** < 0.0001). Data from one of 2 representative donors is presented.

Supplementary Figure 9 Percentage of giant OCs (within the total number of OCs). Giant OCs make up a significant proportion of total OCs. As all treatment conditions were well below 50%, alterations in total OC number are mostly due to changes in the number of small OCs. Each data point represents the percentage of giant OCs of the total OC number, analyzed in a square of 1 mm^2^, of which 6 were assessed per culture well. Columns show mean and standard deviation. Asterisks indicate level of significance in comparison to CO, set at *P* < 0.05 (* < 0.05, ** < 0.01, *** < 0.001, **** < 0.0001). All data is acquired from at least 2 independent experiments, each with a minimum of 3 donors.

Supplementary Table 1 Checkpoint modulators, final concentrations, and suppliers used to investigate their effects on OC differentiation.
